# Metal Particles Are Inappropriate for Testing a Postulate of Extrapulmonary Transport

**DOI:** 10.1289/ehp.115-1817712

**Published:** 2007-02

**Authors:** Andrew J. Ghio, William D. Bennett

**Affiliations:** Office of Research and Development, U.S. Environmental Protection Agency, Research Triangle Park, North Carolina, E-mail: ghio.andy@epa.gov; Center for Environmental Medicine, Asthma, and Lung Biology, University of North Carolina, Chapel Hill, North Carolina

Exposure to ambient air pollution particles has been associated with increased human morbidity and mortality, much of which is nonpulmonary. One proposed explanation of this extrapulmonary tissue injury is a transport of the particles outside of the respiratory tract. In the August 2006 issue of *EHP*, Elder et al. (2006) tested a postulate of extrapulmonary transport of particulate matter (PM). Specifically, the authors focused on the potential translocation of particles by olfactory neuronal pathways to the central nervous system. Comparable to previous studies on systemic transport of PM, they used a metal particle (i.e., a manganese oxide). Elder et al. measured tissue Mn concentrations in an effort to establish transport of the particle.

Past research has repeatedly demonstrated that components of PM can be solubilized, mobilized, and transported to tissues outside the respiratory tract independently of the original particle (e.g., nicotine transport from the cigarette-smoke particle to the blood and central nervous system of the smoker). Metals are among those components that can be solubilized and mobilized from particles and distributed systemically without the translocation of the particle from the original site of deposition in the respiratory tract. To meet the demands of growth and homeostasis, living systems frequently acquire metals from particles using some combination of chemical reduction and direct chelation (Currie and Briat 2003). Elder et al. (2006) reported that the transport of Mn, with elevated concentrations of the metal in extrapulmonary tissues, supports a translocation of the original PM into the central nervous system. However, their use of Mn oxide to test any postulate of extrapulmonary transport of a particle is inappropriate as a result of the *in vivo* availability of both reductants (e.g., superoxide, ascorbate, glutathione) and chelators (e.g., transferrin, lactoferrin, citrate, urate) in the mammalian respiratory tract. These reductants were not available in their *in vitro* solubility tests (performed in normal saline), the results of which they used to support their conclusion of direct *in vivo* translocation of particles. Elevated concentrations of the metal in extrapulmonary tissues do not prove direct translocation of the original PM, but rather reflect solubilization and mobilization of the Mn from the oxide particle, with subsequent distribution. Additionally, the rapidity of change in metal concentration at an extrapulmonary site should not support a direct translocation because the time required for solubilization, mobilization, and systemic transport of a metal from any particle has never been defined; the required time for such transport is predicted to be short for ultrafine particles, which have an increased surface area available for rapid interactions of the PM with endogenous reductants and chelators. What is required to prove the existence of an extrapulmonary transport of PM is employment of a particle with components that cannot be independently solubilized, mobilized, and transported from the site of its original deposition. A carbon-based PM would be appropriate.

When evaluating previous investigations for evidence of extrapulmonary transport of carbon-based particles, there is little to support translocation of such PM outside the respiratory tract. A recent study by Mills et al. (2006) showed no translocation of insoluble, radiolabeled ultrafine carbon particles from the lung into the bloodstream of humans. The small amount of radioactivity they found in the bloodstream and other organs within 6 hr of inhalation could be attributed entirely to the small amount of leached, soluble radiolabel from the particles. Among miners who were exposed to coal dust at 1,000 times the concentrations of ambient air pollution PM, LeFevre et al. (1982) observed the particle in only the lung and the reticuloendothelial system; the presence of the particle in the reticuloendothelial system reflects the transport of PM after sequestration within phagocytes, because the particles are always detected within these cells at these sites.

Finally, decades of research have provided no evidence of an extrapulmonary transport (including via olfactory neuronal pathways) of particles associated with cigarette smoking; smoking exposes the individual to literally kilograms of a particulate combustion product that includes ultrafine particles. While again demonstrating that a metal component of a particle can be solubilized, mobilized, and transported from the site of original deposition, Elder et al. (2006) provided no evidence to support a disbursal of the actual PM to tissues outside the respiratory tract.
